# What Do You Want to Eat? Influence of Menu Description and Design on Consumer’s Mind: An fMRI Study

**DOI:** 10.3390/foods10050919

**Published:** 2021-04-22

**Authors:** Diego Gómez-Carmona, Francisco Muñoz-Leiva, Alberto Paramio, Francisco Liébana-Cabanillas, Serafín Cruces-Montes

**Affiliations:** 1 Department of Marketing and Communication, Faculty of Social Sciences, University of Cadiz, 11405 Jerez de la Frontera, Spain; alberto.paramio@uca.es (A.P.); serafin.cruces@uca.es (S.C.-M.); 2 Instituto de Desarrollo Social y Sostenible (INDESS), University of Cadiz, 11405 Jerez de la Frontera, Spain; 3 Department of Marketing and Market Research, Faculty of Business and Economics, University of Granada, 18071 Granada, Spain; franml@ugr.es (F.M.-L.); franlieb@ugr.es (F.L.-C.); 4 Sport and Health University Research Institute (iMUDS), 18016 Granada, Spain; 5 Department of Psychology, Faculty of Education Sciences, University of Cadiz, 11519 Puerto Real, Spain

**Keywords:** restaurant dish, design dish, description, neuromarketing, emotion, pleasant, fMRI

## Abstract

The main objective of this research was to analyse the active regions when processing dishes with a pleasant (vs. unpleasant) design and the effect of the previously read rational (vs. emotional) description when visualising the dish. The functional magnetic resonance image technique was used for the study. The results showed that participants who visualised pleasant vs. unpleasant dishes became active in several domains (e.g., attention, cognition and reward). On the other side, visualisation of unpleasant dishes activated stronger regions linked to inhibition, rejection, and related ambiguity. We found that subjects who read rational descriptions when visualising pleasant dishes activated regions related to congruence integration, while subjects who visualised emotional descriptions showed an increased neuronal response to pleasant dishes in the regions related to memory, emotion and congruence.

## 1. Introduction

### The Relevance of the Presentation of the Dish in a Restaurant

The restaurant menu is sometimes the first way a restaurant can communicate with potential customers. The information about each menu dish shows clues to the potential customer about the upcoming dining experience [1]. This information will help the consumer to imagine the shape, flavour, or taste of the food [2]. The way the menu content is presented by restaurants can determine the type of restaurant and its personality, even the brand image (ecological, luxury, minimalism or fast food) [3]. If the information conveyed about each dish in the restaurant menu is well designed, this could turn attention towards those more profitable dishes, which would facilitate their sale [4]. Supporting this textual information with images is vitally important because design and aesthetics are among the most valued attributes when choosing a consumer product [5], such as a meal. The relevance for consumers of a food’s attractiveness and appetite has led some researchers to study its influence on decision-making [6]. Some studies have analysed the forms of food, the design of menus, and the behaviour of consumers when faced with different stimuli [2,7,8]. For example, when the same food is served in different coloured dishes, the food sensory and hedonic perception changes [8]. Along with these attributes, other studies have analysed the designation of origin and the complexity or ambiguity of the words and the images used to design the menu using self-reporting techniques [4,7]. Eye-tracking metrics have also been used to test customer responses and the impact of dish placement on the menu [9]. Most consumers feel the need to know what the restaurant dishes look like at the price before deciding. The information available on the menus helps customers to compare dishes from different restaurants before deciding what to eat [10].

Cognitive learning theory suggests that presenting information in a multimodal format can establish a more positive attitude than when a unimodal format is used [1]. According to this theory, diners tend to choose more of a dish from the menu when the picture is detailed together with a description, instead of presenting only the description [1,11]. Therefore, it seems fundamental for research and restaurant management to understand the importance of aesthetics, the design of the dishes, and the name used, because these aspects influence the consumer’s mind [12]. To date, the effects generated by a pleasantly designed dish and its description have not been sufficiently evaluated.

This study aims to analyse the process before the consumption of a restaurant dish. We used neuroimaging to explore the processing of information underlying this moment. In particular, we analysed the regions that anticipate reward or loss, after reading a restaurant menu with rational (vs. emotional) descriptions. We explored the neural nature of the impact of pleasant (vs. unpleasant) design. We also examined the brain areas that could anticipate a greater attitude towards the dish and those areas involved in the final selection of the dish.

## 2. Literature Review

### 2.1. Neural Correlates of Pleasant Dishes

The vision of a pleasant dish (e.g., on a restaurant menu) can be a primary reward and generate positive expectations. In other words, the expectation of valuable rewards can be transferred through the visualisation of a delightful dish. A shared set of brain areas involved in the processing of pleasure has been detailed in neuroscience studies, just as expectations of the dish to be consumed imply the anticipation of positive rewards [13,14,15]. Bartra et al. [16] carried out a meta-analysis on the areas commonly involved in value coding and sensitivity to rewards. In their research, the previous authors examined the neural correlates of subjective value. They found that the ventral striatum and posterior cingulate cortex are strongly implicated in the positive effects of the reward domains.

The authors also advanced that rewards positively stimulate the back of the brain (brain stem). Studies exploring the neural processing of rewards in food-obese participants support brain stem function related to reward [17]. In healthy subjects, the ventral and dorsal strata are the most active [18]. Alongside these rewarding regions, the left fusiform gyrus, left hippocampal gyrus, right amygdala, left caudate nucleus, right putamen, and bilateral cingulate gyrus are active to a lesser extent. At the same time, several studies have shown that dieters tend to visualise their favourite foods [19]. Visualising or smelling very tasty or very sweet food has strong effects on the brain regions associated with reward [19,20]. In the same vein, participants who visualised unhealthy foods found neuronal responses in regions such as the insula, medial and superior frontal gyrus, the inferior right frontal gyrus, inferior left parietal lobes and postcentral gyrus [21]. Subjects with regular vs. restricted sleep also found positive activity in areas of arousal such as the thalamus, precuneus, and medial cingulate gyrus. Considering the previous studies defining the active regions when processing rewards, we propose the following research question:

RQ1: Which regions will be active in processing pleasant dishes?

### 2.2. Neural Correlations of Unpleasant Dishes

Food rejection is one of the most primitive responses of human disgust [22]. On the evolutionary basis of food rejection, we believe that images of unpleasant dishes (e.g., poorly presented dishes) will be a very appropriate stimulus for analysing individual differences in the correlates of disgust, loss, or rejection. Understanding these regions when designing dishes may be of interest to marketing managers due to their influence on purchase intent.

The use of unpleasant dishes provides an understanding of the neural basis for food rejection. This prior attitude can determine future behaviour, especially in more sensitive individuals. Some studies link the neural correlations related to ambiguity, loss, repulsion and negative emotions with a lack of choice [23,24]. Consequently, a negative value likely to be transmitted during the presentation of an unpleasant dish may activate brain regions related to satiety, inhibition and memory, a combination that facilitates food regulation [25]. Among other regions involved in lower-level processing of appetite stimuli, the cerebellum and middle occipital gyrus have been widely associated with danger [26] and punishment [16]. Becker et al. [27] found a positive association between these stimuli and inferior occipital region activity during the processing of rotten food images. For example, Lane et al. [28] used unpleasant vs. neutral images and detected activity in the hippocampus, bilateral occipital cortex, cerebellum, left parahippocampal gyrus, and amygdala. This same visualisation paradigm of unpleasant images activates the bilateral cerebellum and bilateral occipital lobe [29].

In general, evidence points to relationships between the above regions and peripheral and central processing of food-related signals supporting the control of food intake [30]. Visual food-related signals associated with negative consequences can influence sub-sequent eating behaviour. Hence, the importance of plate presentation in eating behaviour [31].

Derived from the above approaches, we propose the following research question:

RQ2: Which regions are activated when processing unpleasant dishes?

### 2.3. Neural Correlates of Emotional Description

Current lines of research combine food science, marketing and psychology. This new multidisciplinary approach allows emotions to be measured jointly [32] by triangulating behavioural data collected through self-report with physiological measures and facial expressions [33]. Information from physiological recordings provides insight into biological responses derived from an emotion (e.g., heart rate, respiratory rate, and electrodermal responses). Furthermore, it is possible to measure emotions by analysing the ocular response [34]. Together with these measures, it is possible to measure neural activity by electroencephalogram or by neuroscientific techniques.

Consumer neuroscience analyses the emotional responses derived from smells and tastes, trying to find the emotional mechanisms that a plate of food can evoke [35]. The main findings show that the emotional valence of the smell (pleasant or unpleasant) is encoded in the orbitofrontal regions [36,37,38], while intensity (the other emotional component of smell) is encoded in the amygdala [39]. In addition to smell, the effect that the taste of food has on the consumer is another factor well documented in the literature. The work of Wilk et al. 2014, explored the tastes of five beverages through physiological and behavioural techniques, analysing the response of the autonomic nervous system of a group of subjects, while recording the heart rate, skin conductance, and temperature of the skin.

Another aspect that can influence the emotion generated by a dish is the description of the dish. Although a priori, it may seem that the pleasure of consuming a dish depends solely on its molecular composition and the individual’s level of hunger. This is not always the case. The influence of marketing actions, such as description, can influence the pleasure experienced [40]. The description of the dish indicates what can be expected from the meal afterwards. The sheer exuberance of the description somehow adds to the appeal of the food described. Furthermore, implicitly, the description can influence many social, cognitive, and affective aspects that combine to guide food preference. Behavioural evidence suggests that cultural messages can generate choices for one consumable or another. Thus, the appeal or dislike of culturally relevant images and associated memories contribute to the modern construction of food preferences [41]. It is normal to think that we know what we like, but we are very influenced by these messages. When a consumer is in front of a restaurant menu, it is usual for them to visualise the description of the dishes until they find the one that meets their expectations [7]. People evaluate the description and can compare it to their beliefs about the fundamental characteristics of the dish. The expectations created by the description can sometimes bias the assessment of taste unless they are entirely refuted [42]. The work of Plassmann et al. [40] showed that the manipulation of the price of different wines influences the reward experienced by subjects. Laboratory outcomes show that the post-consumption review seems to be generally assimilated to previous expectations [7]. If you think it will taste good, it will probably taste good. If you think it will taste bad, it will probably taste bad. Therefore, the mental image which is made after reading the description of the dish can generate expectations in the consumer that can be matched by looking at the dish if the image satisfies the consumer. In this case, when processing the information, the active regions will be linked to rewards, pleasure, and congruence, derived from a satisfactory resolution of the conflict. Congruent stimuli are often described as more enjoyable or rewarding, especially in the context of food products [6]. The description of the dish on display at any given time may anticipate the reward that will be obtained from tasting the dish; the prediction of rewards on different time scales has been studied previously [43]. In a restaurant, we can easily select the dish we will eat from those on the menu. This choice provides the greatest value at a time before consumption. At that time, we compare the expected rewards from other dishes, just based on the description.

The stimulus–action–reward association has shown that subjects strongly activate the dorsal striatum when selecting their food preferences [44]. The dorsal striatum is a region formed by the caudate nucleus and lenticular nucleus. According to Bear, Connors and Paradiso [45], the lenticular nucleus is composed of the putamen and globus pallidus. Activity in these regions is related to the anticipation of gains in tasks that do not involve learning behaviour (i.e., stimulus–reward association) [46]. Several studies have confirmed that prior information about a reward (e.g., description of a dish) makes it possible to predict the reward received from that information [47]. Different learning models through positive reinforcement explain this temporal anticipation of rewards [48]. Learning models suggest that the prediction of reward, whether dependent or independent of action, is learned at the dorsal level [49,50]. Specifically, the putamen is mainly concerned with evaluating actions according to sensory contexts and rewards [43].

In addition to these regions, other brain areas are responsible for the delivery of rewards. In different studies where primary rewards are present (e.g., pleasant taste, touch or smell) the brain activates the insula, anterior cingulate and striatum [38,51]. Using fMRI, Westen et al. [52] found activity in the bilateral caudate, bilateral putamen and pallidum. The authors indicate that in paradigms where subjects experience contingencies between behaviour and reward, it is common for subjects to feel great relief once the situation that caused the risk of cognitive-emotional conflict has ended. Overall, this seems to indicate the existence of a system of motivational reinforcement.

Knutson et al. [53] extended the knowledge of the brain’s response to gains and losses, including the reaction time of subjects. According to the task’s performance, the authors found significant activation in the striated regions (caudate and putamen) according to the performance of a task. In uncertain environments that require conflict resolution, the bilateral caudate and left ventral striatum are usually active. The ventral striatum is activated when the reward is shown; furthermore, it is possible to identify the brain areas that respond to financial reward. These are the ventral striatum and subthalamic midbrain areas [54]. It is also possible to identify those regions that respond to increased reward; specifically, the globus pallidus and cingulate regions [54]. In general, active regions seem to imply a higher order of reasoning. This reasoning will allow the decision-maker to discern which of the options presented is the best before making a decision.

### 2.4. The Neuronal Correlates of Rational Description

We have little knowledge about the relational thinking that integrates the combination of the description of a dish with a pleasant (vs. unpleasant) image of it. How consumers process the relationship by connecting the descriptions on a restaurant menu with the dishes offered is an important and still an unclear marketing issue. Relational reasoning is high-level associative thinking; therefore, processing the consciousness of a rational description of a restaurant dish requires high-level reasoning skills about its relevance and meaning in a given context based on spatial and linguistic processes [1].

Relational thinking allows us to temporarily bring together distant pieces of information through a cognitive process that seeks to connect them. This process consists of completing a logical vacuum that mitigates the lack of information about the present scenario; thus, the brains of consumers reduce ambiguity and achieve certain psychological stability.

When subjects apply relational thinking, it makes it easier to join isolated pieces of information and complete the meanings of the contexts. It is possible to find examples of relational thinking when we analyse analogies such as “food is to restaurant, as an article is to the journal”. The last result of this relational thinking allows subjects to reach conclusions derived from a deductive reasoning process [1]. Applying this form of reasoning, consumers who find themselves at a restaurant table and see the description of a dish for the first time will try to give meaning to this information and form a mental image by joining the parts of information that they already know.

Neuroimaging showed the neural correlates linked to relational thinking using fMRI [55]. The results suggest that relational thinking is the development or process used by our brain to reasonably form a reality about which we know some information. Additionally, linguistic and spatial processes can be the basis of deductive reasoning [56]. These linguistic processes activate the lingual gyrus, usually in response to actions that require reasoning (e.g., “all dishes have potatoes; chicken breast is a dish; therefore, chicken breast has potatoes”). When subjects are faced with situations that require abstract thinking, the parietal brain regions appear to be active in shaping that thinking, namely, the superior parietal lobe [1]. Neuroscience has demonstrated the parietal lobe activity in the coding of relational spatial information [57,58]. In particular, the superior parietal lobe has been shown to play an important role in the orientation and spatial location of visual signals [58]. Furthermore, this region is related to directed attention [59].

It seems that to recognise a plate by its rational description will be necessary to involve brain mechanisms that activate regions linked to the processing of spatial and linguistic localisation. The description of a plate can makes us imagine it and activate spatial processing. From the information obtained in the description, it is possible to give meaning to what is read and activate linguistic processing.

Considering the above and following Heider’s Balance Theory, we can anticipate that a restaurant dish presented after visualising a description will activate relational thinking mechanisms as a consequence of the previous information offered by the description. The subject receives initial pre-persuasive information from the restaurant menu (element 1); the description of the dish itself increases the receptivity of the subject towards a specific dish (element 2). In this initial stage of the process, the subject reads the dish’s name, establishing a relationship between element 1 and element 2. This change will lead to a second stage where the subject will vary their choice to maintain the harmony of cognitive mechanisms, excluding changes that might affect their psychological stability. This leads us to think that identifying a plate is a logical and multifaceted process that activates different brain regions, among which are the areas linked to linguistics, such as the lingual gyrus, and other regions linked to the spatial location (such as the superior parietal lobe). According to the previous literature, we consider that the integration of description and congruent visual stimuli is a different process compared to the interpretation of description and incongruent visual stimuli. The lack of previous studies inclines us to consider the following research questions:

RQ3: Which brain regions are activated when processing emotional descriptions and visualising pleasant dishes?

RQ4: Which brain regions are activated when rational descriptions are processed and pleasant dishes are visualised?

## 3. Methodology

### 3.1. Data Collection and Fieldwork

The fieldwork was carried out at the Centre for Research on the Mind, Brain and Behaviour (CIMCYC) at the University of Granada, Spain. Data were obtained from six women and six men between 25 and 35 years of age. All of them were skilled and healthy, and most of them were undergraduate students (83.3%) with an income of over EUR 1300 per month.

Participants were contacted through mobile phone and email. They were informed about the aims of the study and the characteristics of the experiment. Before the exploration, the participants declared they did not follow any food restrictions and reported no food allergies.

All participants also gave prior written consent. The study was approved by the Ethics Committee of the University of Granada following the stipulations of the World Medical Association (WMA) Declaration of Helsinki (2013).

### 3.2. Stimuli and Experimental Design

A group of 24 students evaluated a set of images using a Likert scale, where 1 was very unpleasant and 5 was very pleasant. Once the average evaluations of all images were determined, we selected 12 images of pleasant dishes (PD) with an appealing design (mean = 4.25) and 12 images of unpleasant dishes (UD) with an unappealing design (mean = 2.75). A group of seven experts who were part of the research team classified the descriptions as emotional or rational.

**Figure 1:**
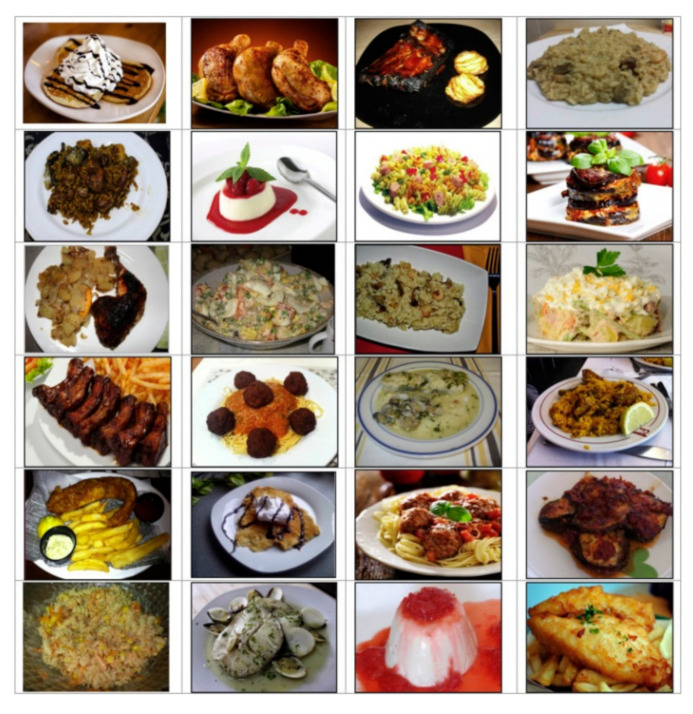

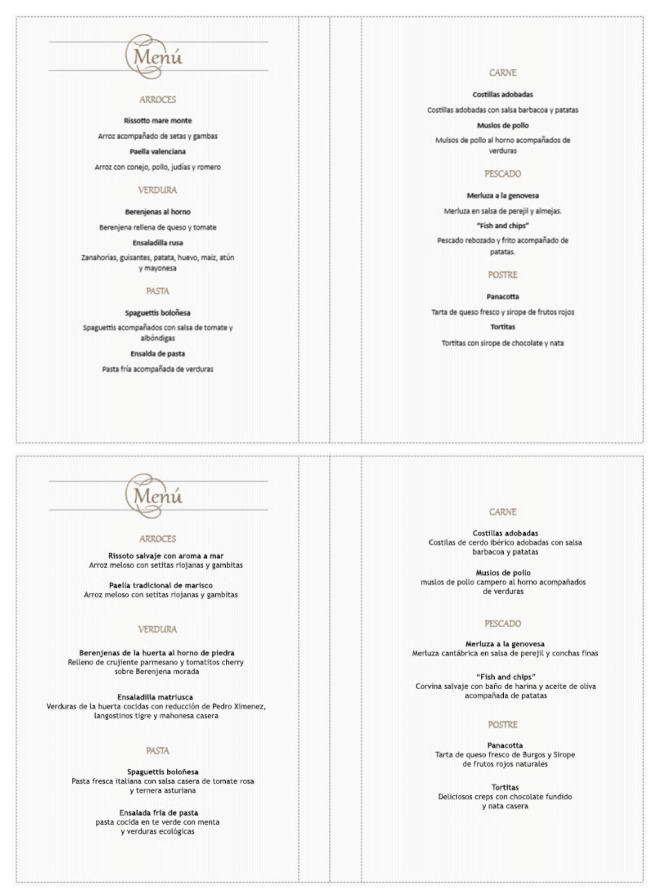
Dishes and menu used.

Before the MRI test, we gave half of the participants a menu with emotional descriptions (ED) of the dishes (e.g., “mellow rice with Riojan mushrooms and prawns”). The other half of the participants read a menu with rational descriptions (RD) of the dishes (e.g., “rice with mushrooms and prawns”). We showed the stimuli three times while scanning the subjects (Figure 1).

We randomised the images using E-Prime 2.0 (Psychology Software Tools, Pittsburgh, PA, USA). The experimental design was made up of four levels or factors, coming from the four conditions (PD × ED, PD × RD, UD × ED and UD × RD). These factors corresponded to a pleasant/unpleasant design and an emotional/rational description), giving rise to four experimental conditions.

**Figure 2:**
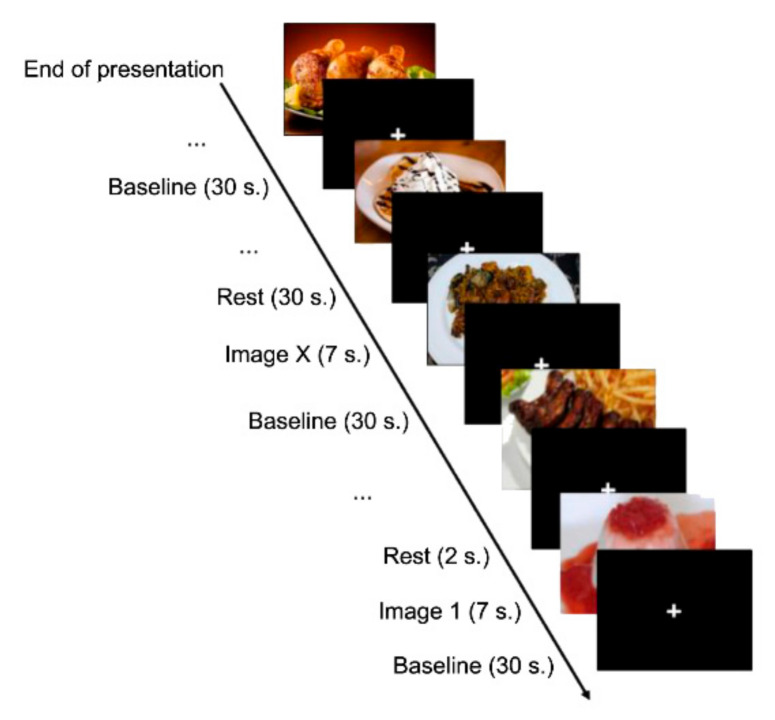
Experimental design.

Once inside the MRI scanner, subjects viewed each image for seven seconds. In addition, we included 72 intervals formed by a black background and a white cross in the centre among the images. We also included a baseline of 30 s duration with the same shape as the stimuli between intervals and between each of the three exposure periods; therefore, the experiment lasted 1098 s (Figure 2).

### 3.3. Procedure for the Acquisition and Pre-Processing of fMRI Scans

The study consisted of one session. We called the participants to the research centre 45 min before starting the MRI task. We informed each participant of the procedure to be followed before entering the fMRI scanner. The fMRI scan procedure consisted of localisation, structural, and functional scans. While the scanner extracted the functional images, the subjects performed the passive viewing task.

The scanner used was a Magneton Prisma Fit 3T (Siemens Healthcare, Madrid, Spain) provided with software for acquiring anatomical and functional images of the brain. We used a gradient echo sequence technique for enhancement in T1 (1 mm→1 mm→1 mm voxels) and T2 * (64→64→3 mm pixels, TE = 35 milliseconds). This is how the blood-oxygenation-level-dependent (BOLD) images were obtained. Employing 3 mm axial sections, we visualised the whole brain with 2 mm thick echoplanar images. We obtained a cut every 90 milliseconds. This meant that 37 cuts for each volume were obtained. We only needed one acquisition period to record all the data. Additionally, we used a repetition time (RT) of 3 s per volume to acquire the 337 images from each session.

Once the exploration was over, the subjects were moved to an adjacent room. They were asked about their attitude towards different dishes.

We used MATLAB R2015b (MathWorks, Sherborn, MA, USA) to pre-process the fMRI data. Specifically, we applied the statistical parametric mapping package (SPM V12). To avoid finding magnetic field saturation effects, we discarded the first seven slices before pre-processing the images. Through interpolation, we realigned the functional images [60]. These images were then spatially normalised following the pattern set by the Montreal Neurological Institute (MNI). Finally, we stabilised the BOLD signal and smoothed the images using a Gaussian core of 8 mm maximum-medium width. In this way, we recorded the movement parameters based on the general linear model.

### 3.4. Whole-Brain Analysis

The contrasts generated in each subject during the first-level analyses were: pleasant (PD × RD + PD × ED) vs. unpleasant (UD × RD + UD × ED) and their inverse. For the contrasts made to the group during the second level of analysis, two-sample *t*-tests were carried out as follows: emotional description (PD × ED) vs. rational (PD × RD). The group size threshold was established by applying the cp cluster Pthresh function. This function provides the minimum cluster size (in voxels) corrected by familywise error (FWE). In addition, it allows for obtaining a significant *p*-value < 0.05, which is ideal for multiple whole brain comparisons [61]. This allowed us to require an uncorrected *p*-significance level < 0.001. Given the exploratory nature of the analyses, we set a minimum cluster size between 10 and 15 voxels.

## 4. Results

### 4.1. Attitudinal Outcomes

The IBM Statistical Package for the Social Sciences (IBM SPSS Statistics, Version 20.0. Armonk, NY, USA) statistical software was used to assess whether there were differences in attitude towards the chosen dish between consumers who were shown the menu with emotional descriptions and those who were shown the menu with rational descriptions. A Kruskal–Wallis test revealed significant differences between the attitudes of consumers towards the dishes (Z = −2.939; *p* = 0.03). In particular, subjects who read the emotional descriptions (mean = 5, SD = 0) had a higher attitude towards the dishes. The scores towards the dishes were lower in those who read the rational descriptions (mean = 3.33, SD = 0.516).

### 4.2. Pleasant and Unpleasant Design Contrast

**Figure 3:**
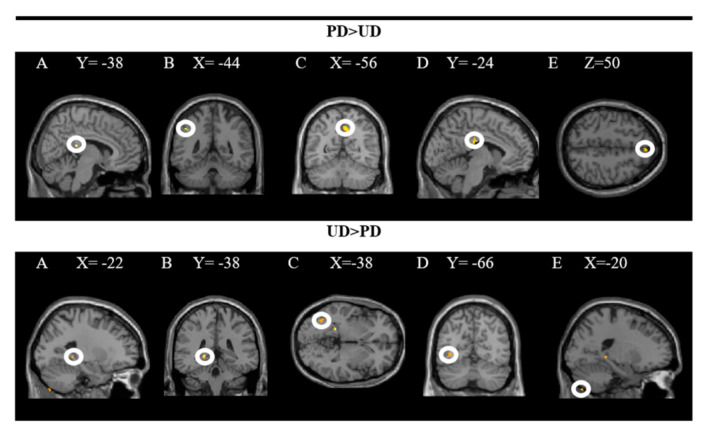
Brain regions activated more strongly to pleasant vs. unpleasant dishes (PD > UD) and to unpleasant vs. pleasant dishes (UD > PD).

The groups in the rear R cingulate gyrus, L inferior parietal lobe, R pre(cuneus), L middle cingulate gyrus, and R superior frontal gyrus were activated more strongly when comparing the pleasant (PD × RD + PD × ED) and the unpleasant (UD × RD + UD × ED) plates. In contrast, the L hippocampus, L inferior occipital gyrus, and L exterior of the cerebellum were activated more significantly when comparing unpleasant to pleasant dishes. In these analyses, a significance level of *p* = 0.001 was established without correction. The results are presented in Figure 3 and the coordinates of the peak are in Table 1.

The upper part of the figure shows a T-map with a threshold of *p* < 0.0001 uncorrected for multiple comparisons (T > 3.2), which is superimposed on the mean anatomical image of all subjects (NIM-space). A: R posterior cingulate gyrus; B: L inferior parietal lobe; C: R precuneus; D: L middle cingulate gyrus; E: R superior frontal gyrus. The lower part shows an uncorrected threshold T map at *p* < 0.0001 for multiple comparisons (T > 4.5), which is superimposed on the mean anatomical image of all subjects (MNI-space). A and B: L hippocampus; C and D: L inferior occipital gyrus; E: L outer cerebellum. See the corresponding coordinates of the peaks in Table 1.

### 4.3. Contrasting Emotional and Rational Descriptions

**Figure 4:**
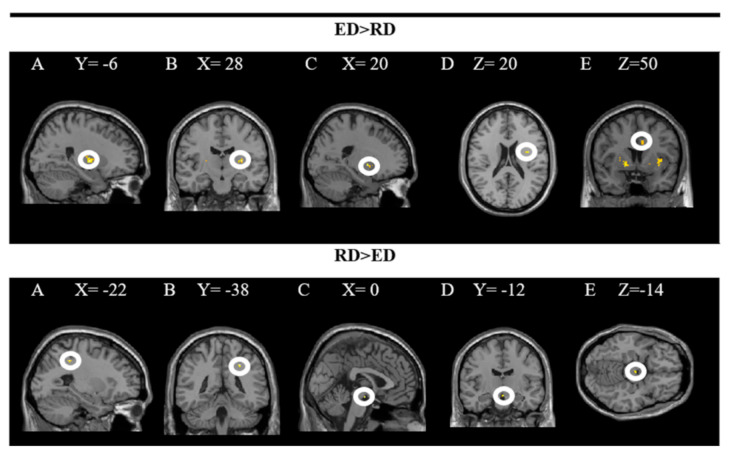
Brain regions activated more strongly to emotional vs. rational description (ED > RD) and rational vs. emotional descriptions (RD > ED).

The findings showed significant activity in the following areas: L putamen, R putamen, R pallidum, R insula and R middle cingulate gyrus while processing dishes with emotional descriptions (ED × PD). The results of the inverse contrast (RD × PD) indicate greater activity in the R superior parietal and ventral DC when the dishes on the restaurant menu with a rational description (vs. emotional) are processed. In these analyses, a significance level of *p* = 0.005 was established without correction. The results are presented in Figure 4 and the coordinates of the peak are in Table 2.

The upper part of the figure shows a T-map with a threshold of *p* < 0.0001 uncorrected for multiple comparisons (T > 2.73), superimposed on the average anatomical image of all subjects (NIM-space). A: L putamen; B: R putamen; C: R pallidum; D: R insula; E: R middle cingulate gyrus. The lower part shows a T map with a threshold of *p* < 0.0001 uncorrected for multiple comparisons (T > 3.2), superimposed on the average anatomical image of all subjects (MNI-space). A and B: superior parietal R; C, D and E: ventral DC. See the corresponding coordinates of the peaks in Table 2.

## 5. Discussion

To this day, no research in hotels and restaurants has examined the neural correlates and behavioural reactions to specific menu items and their descriptions. To the best of our knowledge, this is the first research to explore the neuronal nature of the combined effect of dish design and description on a menu with neuroimaging and self-reporting tools.

In general, the results showed that pleasant and unpleasant design combinations activate different regions at the brain level and also generate different attitudes, according to previous research on the effects of combinations [2]. Findings revealed more positive attitudes towards emotionally described dishes compared to rationally described dishes.

### 5.1. Combination of Descriptions and Pleasant Dishes

Neural correlates explain the nature of a greater attitude towards pleasantly designed dishes. Combinations of pleasing dishes and emotional descriptions result in an increased activation of primary visual regions, which is associated with increased attention to these dishes. On the other hand, unpleasant dishes with rational descriptions activate areas linked to the processing of rejection and satiety most strongly.

More precisely, brain areas identified in previous studies involved in integrating rewards, PD (namely, the ventral striatum, cingulate cortex, superior frontal gyrus, pre(cuneus) and middle cingulate gyrus), are more strongly activated in response to pleasant combinations than to unpleasant combinations.

The description of dishes and their design can convey positive rewards that whet the appetite and therefore lead to the activation of neural patterns involved in the processing of rewards. Hubert et al. [62] discovered the neural correlates associated with reward and pleasure. These brain regions can be activated by visualising an enjoyable dish, anticipating the pleasure derived from tasting a reward similar to the dish shown in the image [63]. Bartra et al. [16], after performing a meta-analysis of reward signal processing concluded that the brain areas involved in processing subjective value (e.g., a primary reward such as food) are located in areas close to the ventromedial prefrontal cortex (such as the middle or superior frontal gyrus) and cingulate gyrus. However, previous studies looking at primary rewards used food directly. Our study results showed that these areas are also activated in response to food images when the dishes are pleasurable. Previous studies on the neural processing of rewards showed that the activation of areas involved in value (such as the posterior gyrus cingulate or pre(cuneus) are the result of the retrieval of episodic memories, linked to attractive dishes [2]. Activity recorded in the previous literature associates with the rewards allows us to answer our first research question.

Studies on consumer choices have shown the involvement of the anterior cuneus and inferior parietal gyrus in deciding under risk contexts [64], such as selecting a dish and then it not resulting in what is expected. The meta-analysis by Krain et al. [64] indicates that, along with these regions, the superior frontal gyrus is also involved in the processing of ambiguous information and entails some risk for the decision-maker. These findings are in line with our findings and coincidentally with the work of Casado-Aranda et al. Casado-Aranda et al. [61] showed increased activity in the superior frontal gyrus when participants visualised products accompanied by seals of approval. (i.e., guarantee reward in some way). By showing pleasant dishes, it is possible to activate the inferior front gyrus, which is a region linked to rewarding tasks [65]. Similarly, high-calorie dishes (e.g., a nice dessert) tend to activate the appetite motivational system to a greater extent than low-calorie dishes (e.g., a vegetable dish). The strongest activity in this motivational system is associated with the medial and dorsolateral prefrontal cortex. According to Killgore et al. [66], both regions play a key role in the anticipation of reward [67,68] and the decision to perform an emotional behaviour [69]. In summary, these results suggest that pleasurable dishes accompanied by emotional descriptions result in greater attention paid, recall of past experiences, and higher expected value.

### 5.2. Combination of Descriptions and Unpleasant Dishes

In our study, the areas considered involved in the processing of satiety, inhibition, and negative sensations in previous literature (middle occipital gyrus, hippocampus, cerebellum and amygdala) showed higher activation in response to the visualisation of unpleasant dishes compared to pleasant dishes (analysed together).

The visualisation of unpleasant stimuli further activates areas such as the hippocampus, (a region that can modulate satiety) and helps to control normal eating behaviour [70]. The inferior occipital gyrus was also strongly activated by unpleasant dishes. Previous research has shown that these areas can play a crucial role in inconsistent stimuli [61]. With these regions, offending plates triggered a neural response in the cerebellum, which is a part of the brain involved in sensory attention and motor learning [71]. Their activity shows increased responses to food cues in obese vs. lean individuals [72]. It is thought that the increased activity in the cerebellar region may reflect efforts to regulate eating [73]. The neuronal correlation linked to the production of unpleasant dishes suggests that individuals activate behavioural control regions that slow down the consumption of the dishes shown. We found a stronger activation in the areas of inhibition, rejection, and ambiguity during unpleasant dishes (vs. pleasant dishes), which may reflect a more negative attitude conferred to the dish and therefore to the restaurant and, consequently, to the purchase intention. These findings are of great interest because the neuronal results allow us to ascertain inaccessible differences at a conscious level between the displeasure transmitted by unpleasant dishes and the visualisation of pleasant dishes. Furthermore, it answers our second research question.

Striation is strongly activated at the neuronal level when comparing the subjects who visualised the emotional (vs. rational) descriptions. Several studies reinforce the role of the putamen and pale in processing emotional information relevant to the individual [46]. These regions deal with different functional aspects of learning and link stimulation with the anticipation of reward [43]. Moreover, the regions mentioned are linked to expectations of positive developments in the future [51,74], such as consumption of the dish described. Recent research [75] has concluded that the striated body, insula, and cingulate cortex are associated with reward, hedonic evaluation, attention, and motivation. This finding fits well with this study, answers our third research question, and is consistent with previous literature linking these regions to responses to tasty food images [76], commercial foods [77], cues predicting the subsequent presentation of tasty high-calorie foods [78,79], and the reception of tasty high-calorie foods [80].

Consistent with previous neuroimaging findings, the main effects of relational reasoning between the rational description of the dish and its image elicit activation of the SPL [1] and ventral DC. It has been found that the superior parietal lobe is activated during orientation towards the spatial location of visual signals [58]. In addition, the superior parietal cortex is involved in the ordering, updating, and manipulation of working memory items [81]. It seems that visualising the dish after reading a rational description creates mental logic that activates the codification of relational spatial information. In the case of the more rational and informative and less persuasive descriptions, the visualisation of images manages to direct attention towards pleasant dishes. These results are similar to those achieved in recent studies where congruent (as opposed to incongruent) stimuli were shown [82], although they did not use food stimuli. Furthermore, our results are in line with Chang et al. [83], who observed that SPL activity was detected by showing non-congruent (vs. famous) stimuli.

The above arguments answer our fourth research question and suggest that rational descriptions, when visualising pleasant dishes, activate congruence integration regions. However, it seems that these descriptions do not succeed in transferring emotion to the displayed dish.

## 6. Conclusions

The present study shows the importance of the design of a dish in forming a positive attitude and anticipating the possible rewards that its description may imply. In other words, a good design and a perfect arrangement of the elements of a dish can make it a powerful marketing tool that induces certain ideas related to the pleasure of eating. Therefore, the restaurant menu designer could design the menu using images of pleasing dishes and emotional descriptions that trigger positive emotions in the consumer’s mind at the moment of decision making. It is also possible that the dish’s description or image is reminiscent of a past, pleasurable episode. In the latter case, the customer could quickly process the information, eliciting an emotional response that could condition their choice of a particular dish. Managers should identify the most profitable dishes and display images of them on the menu. This design can convey particular messages to customers, encouraging them to spend more or even make them want to repeat if the experience is good. Our findings also suggest that when we choose a dish to eat in a restaurant, we pay little attention to healthiness, which may influence people to form future eating intentions that are less healthy, and restaurant managers should consider this issue. Therefore, a fair presentation of the menu could improve sales, improve restaurant profitability and improve customer satisfaction, which are the main objectives that every manager ultimately seeks.

In this research, a specific experimental design was developed in order to assess the information processing of the participants. The strict and careful application of the experimental design achieved an improvement in the internal and external validities of the results. However, the results obtained should be treated with caution due to the small sample size. In the future, we hope to replicate this design with a larger sample of individuals to analyse the interactions between all factors, reduce the margin of error, and obtain more consistent and robust results.

The research design itself did not allow us to evaluate the same image of a dish with text and without text. This limitation prevented us from really separating the reward derived from the visual image and the reward provoked by the textual message (emotional or rational). Future research could analyse the impact of different marketing messages (description, price, and even quality seals or designations of origin) on the same image.

Finally, it would be interesting to address the visual relevance of textual vs. visual stimuli in future work. This comparison could be made using eye-tracking methodologies. The information obtained through eye-tracking would allow us to judge the correct placement of descriptions and images of dishes on the restaurant menu.

## Figures and Tables

**Table 1:** Brain regions with stronger activation in response to pleasant (PD × RD + PD × ED) vs unpleasant (UD × RD + UD × ED) dishes.

	Peak MNI-Coordinates MNI			Contrast		
Brain Region	*x*	*y*	*z*	Z	T	Effect Size **
**PD > UD**						
R Posterior cingulate gyrus *	8	−38	18	3.81	5.70	1.09
L Inferior Parietal Lobule *	−44	−46	42	3.41	4.71	0.98
R Precuneus *	8	−56	40	2.98	3.82	0.86
L Middle cingulate gyrus *	−6	−24	26	2.94	3.84	0.84
R Superior Frontal Gyrus *	4	36	50	2.69	3.29	0.77
**UD > PD**						
L Hippocampus *	−22	−38	−2	4.55	8.16	1.31
L Inferior occipital gyrus *	−38	−66	−2	3.40	4.69	0.98
L Cerebellum exterior *	−20	−78	−54	3.32	4.50	0.95

* Peak of clusters significant at *p* < 0.0001 uncorrected, k ≥ 10 voxels are reported. ** Effect Size = Z/√N.

**Table 2:** Brain regions with stronger activation in response to emotional (PD × ED + UD × ED) vs. rational (PD × RD + UD × RD) descriptions.

	Peak MNI-Coordinates MNI			Contrast		
Brain Region	*x*	*y*	*z*	Z	T	Effect Size **
**ED > RD**						
L putamen *	−26	−6	2	3.84	6.05	1.10
R putamen *	28	−14	10	3.68	5.59	1.06
R pallidum *	20	0	−2	3.62	5.42	1.04
R insula *	34	−2	20	3.27	4.55	0.94
R middle cingulate gyrus *	6	6	32	2.73	3.44	0.78
**RD > ED**						
R superior parietal *	26	−42	42	3.70	5.65	1.06
Ventral DC *	0	−12	−14	3.20	4.38	0.92

* Peak of clusters significant at *p* < 0.0005 uncorrected, k ≥ 5 voxels are reported. ** Effect size = Z/√N.

## Data Availability

Data can be requested directly from the authors.

## References

[1] Lee A., Kim M.G. (2020). Effective electronic menu presentation: From the cognitive style and mental imagery perspectives. Int. J. Hosp. Manag..

[2] Muñoz-Leiva F., Gómez-Carmona D. (2019). Sparking interest in restaurant dishes? Cognitive and affective processes underlying dish design and ecological origin. An fMRI study. Physiol. Behav..

[3] Magnini V.P., Kim S. (2016). The influences of restaurant menu font style, background color, and physical weight on consumers’ perceptions. Int. J. Hosp. Manag..

[4] Hou Y., Yang W., Sun Y. (2017). Do pictures help? The effects of pictures and food names on menu evaluations. Int. J. Hosp. Manag..

[5] Zolli A. (2004). Why design matters more. Am. Demogr..

[6] Zellner D.A., Loss C.R., Zearfoss J., Remolina S. (2014). It tastes as good as it looks! The effect of food presentation on liking for the flavor of food A. Appetite.

[7] Wansink B., Love K. (2014). Slim by design: Menu strategies for promoting high-margin, healthy foods. Int. J. Hosp. Manag..

[8] Piqueras-Fiszman B., Giboreau A., Spence C. (2013). Assessing the influence of the color of the plate on the perception of a complex food in a restaurant setting. Flavour.

[9] Reale S., Flint S.W. (2016). The impact of menu label design on visual attention, food choice and recognition: An eye tracking study. J. Sens. Stud..

[10] Brandau M. (2013). Finding Answer for Mobile Search. https://www.nrn.com/blog/finding-answers-mobile-search.

[11] Jang S.S., Kim D. (2015). Enhancing ethnic food acceptance and reducing perceived risk: The effects of personality traits, cultural familiarity, and menu framing. Int. J. Hosp. Manag..

[12] Hagtvedt H., Patrick V.M. (2008). Art infusion: The influence of visual art on the perception and evaluation of consumer products. J. Mark. Res..

[13] Alonso-Alonso M., Woods S.C., Pelchat M., Grigson P.S., Stice E., Farooqi S., Khoo C.S., Mattes R.D., Beauchamp G.K. (2015). Food reward system: Current perspectives and future research needs. Nutr. Rev..

[14] de Macedo I.C., de Freitas J.S., da Silva Torres I.L. (2016). The influence of palatable diets in reward system activation: A mini review. Adv. Pharmacol. Sci..

[15] Segovia M.S., Palma M.A., Nayga R.M. (2019). The effect of food anticipation on cognitive function: An eye tracking study. PLoS ONE.

[16] Bartra O., McGuire J.T., Kable J.W. (2013). The valuation system: A coordinate-based meta-analysis of BOLD fMRI experiments examining neural correlates of subjective value. NeuroImage.

[17] Volkow N.D., Wang G.J., Baler R.D. (2011). Reward, dopamine and the control of food intake: Implications for obesity. Trends Cogn. Sci..

[18] Volkow N.D., Wang G.J., Fowler J.S., Logan J., Jayne M., Franceschi D., Wong C., Gatley S.J., Gifford A.N., Ding Y.S. (2002). Nonhedonic? Food motivation in humans involves dopamine in the dorsal striatum and methylphenidate amplifies this effect. Synapse.

[19] Pelchat M.L., Johnson A., Chan R., Valdez J., Ragland J.D. (2004). Images of desire: Food-craving activation during fMRI. Neuroimage.

[20] Bragulat V., Dzemidzic M., Bruno C., Cox C.A., Talavage T., Considine R.V., Kareken D.A. (2010). Food-related odor probes of brain reward circuits during hunger: A pilot fMRI study. Obesity.

[21] St-Onge M.P., Wolfe S., Sy M., Shechter A., Hirsch J. (2014). Sleep restriction increases the neuronal response to unhealthy food in normal-weight individuals. Int. J. Obes..

[22] Rozin P., Haidt J., Mccauley C.R. (1993). Handbook of Emotions.

[23] Huang Y.F. (2014). Pre-existing brain states predict risky choices. Neuroimage.

[24] Christopoulos G.I., Tobler P.N., Bossaerts P., Dolan R.J., Schultz W. (2009). Neural correlates of value, risk, and risk aversion contributing to decision making under risk. J. Neurosci..

[25] Lowe M.R. (2009). Neural correlates of individual differences related to appetite. Physiol. Behav..

[26] Liddell B.J., Brown K.J., Kemp A.H., Barton M.J., Das P., Peduto A., Gordon E., Williams L.M. (2005). A direct brain- stem–amygdala–cortical “alarm” system for subliminal signals of fear. NeuroImage.

[27] Becker C.A., Flaisch T., Renner B., Schupp H.T. (2016). Neural correlates of the perception of spoiled food stimuli. Front. Hum. Neurosci..

[28] Lane R.D., Reiman E.M., Bradley M.M., Lang P.J., Ahern G.L., Davidson R.J., Schwartz G.E. (1997). Neuroanatomical correlates of pleasant and unpleasant emotion. Neuropsychologia.

[29] Reddy R.P., Korde S.P., Kanungo S., Thamodharan A., Rajeswaran J., Bharath R.D., Upadhya N., Panda R., Rao S.L. (2014). Neural correlates of emotion: Acquisition versus innate view point. Indian J. Psychol. Med..

[30] Higgs S. (2008). Cognitive influences on food intake: The effects of manipulating memory for recent eating. Physiol. Behav..

[31] Kanoski S.E., Grill H.J. (2017). Hippocampus contributions to food intake control: Mnemonic, neuroanatomical, and endocrine mechanisms. Biol. Psychiatry.

[32] Walsh A.M., Duncan S.E., Bell M.A., O’Keefe S.F., Gallagher D.L. (2017). Integrating implicit and explicit emotional assessment of food quality and safety concerns. Food Qual. Prefer..

[33] Lamote S., Hermans D., Baeyens F., Eelen P. (2004). An exploration of affective priming as an indirect measure of food attitudes. Appetite.

[34] Kreibig S.D. (2010). Autonomic nervous system activity in emotion: A review. Biol. Psychol..

[35] Kantono K., Hamid N., Shepherd D., Lin Y.H.T., Skiredj S., Carr B.T. (2019). Emotional and electrophysiological measures correlate to flavour perception in the presence of music. Physiol. Behav..

[36] Zald D.H., Donndelinger M.J., Pardo J.V. (1998). Elucidating dynamic brain interactions with across-subjects correlational analyses of positron emission tomographic data: The functional connectivity of the amygdala and orbitofrontal cortex during olfactory tasks. J. Cereb. Blood Flow Metab..

[37] Rolls E.T. (2001). The rules of formation of the olfactory representations found in the orbitofrontal cortex olfactory areas in primates. Chem. Senses.

[38] O’Doherty J.P., Deichmann R., Critchley H.D., Dolan R.J. (2002). Neural responses during anticipation of a primary taste reward. Neuron.

[39] Anderson A.K., Christoff K., Stappen I., Panitz D., Ghahremani D.G., Glover G., Gabrieli J.D.E., Sobel N. (2003). Dissociated neural representations of intensity and valence in human olfaction. Nat. Neurosci..

[40] Plassmann H., O’Doherty J., Shiv B., Rangel A. (2008). Marketing actions can modulate neural representations of experienced pleasantness. Proc. Natl. Acad. Sci. USA.

[41] McClure S.M., Li J., Tomlin D., Cypert K.S., Montague L.M., Montague P.R. (2004). Neural correlates of behavioral preference for culturally familiar drinks. Neuron.

[42] Richardson G.P., Andersen D.F., Maxwell T.A., Stewart T.R., Wolstenholme E. Foundations of mental model research. Proceedings of the 1994 International System Dynamics Conference.

[43] Haruno M., Kawato M. (2006). Different neural correlates of reward expectation and reward expectation error in the putamen and caudate nucleus during stimulus-action-reward association learning. J. Neurophysiol..

[44] Haruno M., Kuroda T., Doya K., Toyama K., Kimura M., Samejima K., Kawato M. (2004). A neural correlate of reward-based behavioral learning in caudate nucleus: A functional magnetic resonance imaging study of a stochastic decision task. J. Neurosci..

[45] Bear M., Connors B., Paradiso M.A. (2020). Neuroscience: Exploring the Brain.

[46] Tricomi E.M., Delgado M.R., Fiez J.A. (2004). Modulation of caudate activity by action contingency. Neuron.

[47] Takikawa Y., Kawagoe R., Hikosaka O. (2004). A possible role of midbrain dopamine neurons in short- and long-term adaptation of saccades to position-reward mapping. J. Neurophysiol..

[48] Schultz W. (1992). Neuronal activity in monkey ventral striatum related to the expectation of reward. J. Neurosci..

[49] Brown J., Bullock D., Grossberg S. (1999). how the basal ganglia use parallel excitatory and inhibitory learning pathways to selectively respond to unexpected rewarding cues. J. Neurosci..

[50] Montague P.R., Dayan P., Sejnowski T.J. (1996). A framework for mesencephalic dopamine systems based on predictive Hebbian learning. J. Neurosci..

[51] Kirsch P., Schienle A., Stark R., Sammer G., Blecker C., Walter B., Ott U., Burkart J., Vaitl D. (2003). Anticipation of reward in a nonaversive differential conditioning paradigm and the brain reward system: An event-related fMRI study. NeuroImage.

[52] Westen D., Blagov P.S., Harenski K., Kilts C., Hamann S. (2006). Neural bases of motivated reasoning: An fMRI study of emotional constraints on partisan political judgment in the 2004 U.S. Presidential Election. J. Cogn. Neurosci..

[53] Knutson B., Westdorp A., Kaiser E., Hommer D. (2000). FMRI visualization of brain activity during a monetary incentive delay task. NeuroImage.

[54] Elliott R., Friston K.J., Dolan R.J. (2000). dissociable neural responses in human reward systems. J. Neurosci..

[55] Wendelken C., Nakhabenko D., Donohue S.E., Carter C.S., Bunge S.A. (2008). Brain is to thought as stomach is to?: Investigating the role of rostrolateral prefrontal cortex in relational reasoning. J. Cogn. Neurosci..

[56] Goel V., Dolan R.J. (2001). Functional neuroanatomy of three-term relational reasoning. Neuropsychologia.

[57] Laeng B. (1994). Lateralization of categorical and coordinate spatial functions: A study of unilateral stroke patients. J. Cogn. Neurosci..

[58] Fan J., McCandliss B.D., Sommer T., Raz A., Posner M.I. (2002). Testing the efficiency and independence of attentional networks. J. Cogn. Neurosci..

[59] Keuper K., Zwitserlood P., Rehbein M.A., Eden A.S., Laeger I., Junghöfer M., Zwanzger P., Dobel C. (2013). early prefrontal brain responses to the hedonic quality of emotional words—A simultaneous EEG and MEG study. PLoS ONE.

[60] Ashburner J., Friston K.J. (2005). Unified segmentation. Neuroimage.

[61] Casado-Aranda L.A., Liébana-Cabanillas F., Sánchez-Fernández J. (2018). A Neuropsychological study on how consumers process risky and secure e-payments. J. Interact. Mark..

[62] Hubert M., Hubert M., Linzmajer M., Riedl R., Kenning P. (2018). Trust me if you can—Neurophysiological insights on the influence of consumer impulsiveness on trustworthiness evaluations in online settings. Eur. J. Mark..

[63] Riedl R., Hubert M., Kenning P. (2010). Are there neural gender differences in online trust? An fMRI Study on the perceived trustworthiness of ebay offers. Mis Q..

[64] Krain A.L., Wilson A.M., Arbuckle R., Castellanos F.X., Milham M.P. (2006). Distinct neural mechanisms of risk and ambiguity: A meta-analysis of decision-making. NeuroImage.

[65] Rushworth M., Walton M., Kennerley S., Bannerman D. (2004). Action sets and decisions in the medial frontal cortex. Trends Cogn. Sci..

[66] Killgore W.D., Young A.D., Femia L.A., Bogorodzki P., Rogowska J., Yurgelun-Todd D.A. (2003). Cortical and limbic activation during viewing of high- versus low-calorie foods. NeuroImage.

[67] Watanabe M. (1998). Cognitive and motivational operations in primate prefrontal neurons. Rev. Neurosci..

[68] Rolls E.T. (1990). A theory of emotion, and its application to understanding the neural basis of emotion. Cogn. Emot..

[69] Reiman E.M. (1997). The application of positron emission tomography to the study of normal and pathologic emotions. J. Clin. Psychiatry.

[70] Haase L., Cerf-Ducastel B., Murphy C. (2009). Cortical activation in response to pure taste stimuli during the physiological states of hunger and satiety. NeuroImage.

[71] O’Halloran C.J., Kinsella G.J., Storey E. (2012). The cerebellum and neuropsychological functioning: A critical review. J. Clin. Exp. Neuropsychol..

[72] Martin L.E., Holsen L.M., Chambers R.J., Bruce A.S., Brooks W.M., Zarcone J.R., Butler M.G., Savage C.R. (2010). Neural mechanisms associated with food motivation in obese and healthy weight adults. Obesity.

[73] Geliebter A., Benson L., Pantazatos S.P., Hirsch J., Carnell S. (2016). Greater anterior cingulate activation and connectivity in response to visual and auditory high-calorie food cues in binge eating: Preliminary findings. Appetite.

[74] Knutson B., Adams C.M., Fong G.W., Hommer D. (2001). Anticipation of increasing monetary reward selectively recruits nucleus accumbens. J. Neurosci..

[75] Winter S.R., Yokum S., Stice E., Osipowicz K., Lowe M.R. (2017). Elevated reward response to receipt of palatable food predicts future weight variability in healthy-weight adolescents. Am. J. Clin. Nutr..

[76] Demos K.E., Kelley W.M., Heatherton T.F. (2011). Dietary restraint violations influence reward responses in nucleus accumbens and amygdala. J. Cogn. Neurosci..

[77] Yokum S., Gearhardt A.N., Harris J.L., Brownell K.D., Stice E. (2014). Individual differences in striatum activity to food commercials predict weight gain in adolescents. Obesity.

[78] Stice E., Burger K.S., Yokum S. (2015). Reward region responsivity predicts future weight gain and moderating effects of the TaqIA Allele. J. Neurosci..

[79] Yokum S., Ng J., Stice E. (2012). Relation of regional gray and white matter volumes to current BMI and future increases in BMI: A prospective MRI study. Int. J. Obes..

[80] Geha P.Y., Aschenbrenner K., Felsted J., O’Malley S.S., Small D.M. (2013). Altered hypothalamic response to food in smokers. Am. J. Clin. Nutr..

[81] Koenigs M., Barbey A.K., Postle B.R., Grafman J. (2009). superior parietal cortex is critical for the manipulation of information in working memory. J. Neurosci..

[82] Qu F., Wang J., Zhong Y., Ye H. (2018). Postural effects on the mental rotation of body-related pictures: An fMRI study. Front. Psychol..

[83] Chang H.J.J., O’Boyle M., Anderson R.C., Suttikun C. (2016). An fMRI study of advertising appeals and their relationship to product attractiveness and buying intentions. J. Consum. Behav..

